# From phenotype to genotype: an association study of longitudinal phenotypic markers to Alzheimer's disease relevant SNPs

**DOI:** 10.1093/bioinformatics/bts411

**Published:** 2012-09-03

**Authors:** Hua Wang, Feiping Nie, Heng Huang, Jingwen Yan, Sungeun Kim, Kwangsik Nho, Shannon L. Risacher, Andrew J. Saykin, Li Shen, for the Alzheimer's Disease Neuroimaging Initiative

**Affiliations:** ^1^Department of Computer Science and Engineering, University of Texas at Arlington, TX 76019, USA; ^2^Department of Radiology and Imaging Sciences, Indiana University School of Medicine, Indianapolis, IN 46202, USA

## Abstract

**Motivation:** Imaging genetic studies typically focus on identifying single-nucleotide polymorphism (SNP) markers associated with imaging phenotypes. Few studies perform regression of SNP values on phenotypic measures for examining how the SNP values change when phenotypic measures are varied. This alternative approach may have a potential to help us discover important imaging genetic associations from a different perspective. In addition, the imaging markers are often measured over time, and this longitudinal profile may provide increased power for differentiating genotype groups. How to identify the longitudinal phenotypic markers associated to disease sensitive SNPs is an important and challenging research topic.

**Results:** Taking into account the temporal structure of the longitudinal imaging data and the interrelatedness among the SNPs, we propose a novel ‘task-correlated longitudinal sparse regression’ model to study the association between the phenotypic imaging markers and the genotypes encoded by SNPs. In our new association model, we extend the widely used *ℓ*_2,1_-norm for matrices to tensors to jointly select imaging markers that have common effects across all the regression tasks and time points, and meanwhile impose the trace-norm regularization onto the unfolded coefficient tensor to achieve low rank such that the interrelationship among SNPs can be addressed. The effectiveness of our method is demonstrated by both clearly improved prediction performance in empirical evaluations and a compact set of selected imaging predictors relevant to disease sensitive SNPs.

**Availability:** Software is publicly available at: http://ranger.uta.edu/%7eheng/Longitudinal/

**Contact:** heng@uta.edu or shenli@inpui.edu

## 1 INTRODUCTION

Neuroimaging genetics is an emerging research field, where brain imaging is used as quantitative phenotypes to investigate the role of genetic variation in brain structure and function. It holds great promise for a systems biology of the brain to better understand complex neurobiological systems, from genetic determinants to cellular processes to the complex interplay of brain structure, function, behavior and cognition. Disorders of the nervous system are associated with complex neurobiological changes, which may lead to profound alterations at all levels of organization.

Genome-wide association studies (GWAS) have been increasingly performed to correlate high-throughput single-nucleotide polymorphism (SNP) data to large-scale imaging data. To facilitate such association analysis, many studies used a hypothesis-driven approach (Glahn *et al.*, 2007) by making significant reduction in one or both data types. For example, some whole-brain studies focused on a small number of genetic variables (e.g. Brun *et al.*, 2009; Filippini *et al.*, 2009; Hariri *et al.*, 2006; Nichols and Inkster, 2009), and some whole-genome studies examined a limited number of imaging variables (e.g. Baranzini *et al.*, 2008; Potkin *et al.*, 2009; Seshadri *et al.*, 2007). Many SNPs have been identified as risk factors for Alzheimer's disease (AD), see those in the AlzGene database (www.alzgene.org).

So far most studies focus on selecting and associating SNPs to AD status or imaging phenotypes. Very few studies have been done to directly examine how the SNP values change when phenotypic measures are varied, i.e. via regression of SNP values on phenotypic measures. This alternative approach may have a potential to help us discover important imaging genetic associations from a different perspective. In this study, we perform such an initial analysis for finding phenotypic imaging markers that are related to SNPs from or proximal to AlzGene candidates.

Neuroimaging measures have been widely studied to predict disease status and/or cognitive performance (Batmanghelich *et al.*, 2009; Shen *et al.*, 2010a). However, whether these measures coupled with their longitudinal profiles have sufficient power to infer relevant genotype groups is still an under-explored yet important topic in AD research. A simple strategy typically used in longitudinal studies (e.g. Risacher *et al.*, 2010) is to analyze a single summarized value such as average change rate of change or slope. This approach may be inadequate to distinguish the complete dynamics of cognitive trajectories and thus become unable to identify the underlying genetic structure.

With these observations, in this work, we propose a new task-correlated longitudinal sparse regression framework to effectively identify the longitudinal phenotypic markers related to candidate AD SNPs. Based on the emerging structured sparse learning techniques, which has been effectively applied in imaging genetics studies (Wang *et al.*, 2011a, b, 2012a, b), the new combined structured sparse regularizations are introduced to tackle the longitudinal phenotypic patterns and biological genotypic correlations. The proposed new computational biology model consists of three major components. First, due to the serial measures of the imaging phenotypes over time, we propose a novel longitudinal regression analysis method. As a result, the regression coefficients assess the relationships between longitudinal phenotypes and their genetic makeups. Second, certain SNPs are naturally correlated via different ways, e.g. multiple SNPs from one single gene often jointly carry out similar genetic functionalities, SNPs in high linkage disequilibrium (LD) are linked together in meiosis. To incorporate such SNP correlations in our association studies, we propose to use the trace/nuclear norm regularization (Candès and Recht, 2009; Nie *et al.*, 2012) to approximately minimize the rank of regression coefficient matrix, such that the coefficients of phenotypes associated to correlated SNPs are linearly dependent. Finally, through enforcing the *ℓ*_2,1_-norm regularization, the imaging feature selection across most SNPs are coupled (Argyriou *et al.*, 2007; Obozinski *et al.*, 2006), so that the identified imaging phenotypes have common influence on all the SNPs.

We apply the proposed method to the Alzheimer's Disease Neuroimaging Initiative (ADNI) cohort (Mueller *et al.*, 2005) for identifying longitudinal phenotypes using a set of SNPs based on the AlzGene database. Our empirical results yield not only clearly improved prediction performance in all test cases but also a compact set of associations between phenotypes and genotypes that are in accordance with prior research findings.

## 2 MATERIALS AND DATA SOURCES

Both SNP and structural magnetic resonance imaging (MRI) data used in the preparation of this article were obtained from the ADNI database (adni.loni.ucla.edu). One goal of ADNI has been to test whether serial MRI, positron emission tomography (PET), other biological markers and clinical and neuropsychological assessment can be combined to measure the progression of mild cognitive impairment (MCI) and early AD. For up-to-date information, we refer interested readers to www.adni-info.org.

### 2.1 SNP genotypes

The SNP data used in this study (Saykin *et al.*, 2010) were genotyped using the Human 610-Quad BeadChip (Illumina, Inc., San Diego, CA, USA). Among all SNPs, only SNPs, belonging to the top 40 AD candidate genes listed on the AlzGene database (www.alzgene.org) as of June 10, 2010, were selected after the standard quality control (QC) and imputation steps. The QC criteria for the SNP data include (i) call rate check per subject and per SNP marker, (ii) gender check, (iii) sibling pair identification, (iv) the Hardy–Weinberg equilibrium test, (v) marker removal by the minor allele frequency and (vi) population stratification. As the second pre-processing step, the quality-controlled SNPs were imputed using the MaCH software (Li *et al.*, 2010) to estimate the missing genotypes. After that, the Illumina annotation information based on the Genome build 36.2 was used to select a subset of SNPs, belonging to the top 40 AD candidate genes (Bertram *et al.*, 2007). The above procedure yielded 1224 SNPs from 37 genes. For the remaining three genes, no SNPs were available on the genotyping chip.

### 2.2 MRI analysis and extraction of imaging phenotypes

Two widely used automated MRI analysis techniques were used to process and extract imaging genotypes across the brain from all the MRI scans of ADNI participants as previously described (Shen *et al.*, 2010b). First, voxel-based morphometry (VBM) (Ashburner and Friston, 2000) was performed to define modulated gray matter (GM) maps and extract local GM values for target regions. Second, automated parcellation via FreeSurfer V4 (Fischl *et al.*, 1999, 2002) was conducted to define volumetric and cortical thickness values for regions of interest (ROIs) and to extract total intracranial volume (ICV). Further information is available in (Shen *et al.*, 2010b). The time points examined in this study for imaging markers included baseline (BL), Month 6 (M6), Month 12 (M12) and Month 24 (M24). All the participants with no missing BL/M6/M12/M24 MRI measurements were included in this study. Figure 2 shows the names of these ROIs in the brain space. All these measures were adjusted for baseline ICV using the regression weights derived from the healthy control (HC) participants.

## 3 TASK-CORRELATED LONGITUDINAL SPARSE REGRESSION

For the association study of longitudinal imaging phenotypes to the genotypes, the input imaging features are a set of matrices 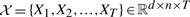 corresponding to the measurements at *T* consecutive time points, where *X_t_* is the imaging measurements for a certain type of imaging markers, such as VBM or FreeSurfer markers used in this study, at time *t*(1 ≤ *t* ≤ *T*). Obviously, 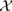 is a tensor data with *d* imaging features, *n* subject samples and *T* time points. The output genetic variations described by *c* SNPs for the *n* subject samples forms a matrix *Y* = [**y**_1_,...,**y***_n_*]*^T^* ∈ ℝ*^n^*^×^*^c^*, where the **y***_i_* ∈ ℝ*^n^*^×^*^c^* is the SNP values of the *i*th subject sample. Our goal is to learn from {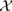, *Y*} a model that can reveal the associations between the longitudinal imaging phenotypes 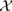 and the genotypes *Y*.

A straightforward method for relating imaging phenotypes and SNPs is to perform regression at each time point separately, which, though, does not take into account the valuable information conveyed by the longitudinal patterns of the phenotypic inputs. To overcome this limitation, different from previous studies that learned the regression coefficient matrix for each time point individually, we aim to learn a unified longitudinal regression model to find the genetic features that are associated to the longitudinal imaging patterns over all the measurement time points. To this end, we expect to learn a coefficient tensor (a stack of coefficient matrices) 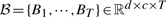 to reveal the temporal changes of the coefficient matrices. In this article, we propose to use the low-rank structured sparse regularizations to explore the temporal patterns and the interrelatedness between SNPs in a new task-correlated longitudinal sparse regression model.

### 3.1 Task-correlated longitudinal sparse regression using low-rank structured sparse regularizations

The simplest model to associate the the phenotypic markers to the genotypes is the multivariate regression model, which solves the following optimization problem:
(1)
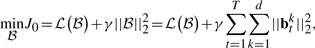

where **b***_t_^k^* denotes the *k*th row of coefficient matrix *B_t_* at time *t*, and 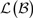 is the proposed longitudinal loss and defined as
(2)
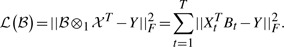

Because the objective *J*_0_ in Equation (1) can be decoupled for each individual time point and does not consider the longitudinal correlations between the imaging features and the SNPs, it is not suitable for longitudinal data analysis and feature selection. Because the selected imaging markers with temporal changes are desired to connect all the SNPs, the *T* groups of regression tasks at different time points should not be decoupled and have to be performed simultaneously. Thus, we introduce the structured sparse regularization (Argyriou *et al.*, 2007; Nie *et al.*, 2010; Obozinski *et al.*, 2006) into the longitudinal data regression and feature selection model as following:
(3)
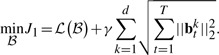

Apparently, *J*_1_ in Equation (3) can no longer be decoupled over time dimension. Upon solution, the imaging features with common influences to all the SNPs across all the time points will be identified out due to the second term in Equation (3), which essentially is a tensor extension of the widely used *ℓ*_2,1_-norm for matrices.

To further take into account that many SNPs are interrelated together and their effects on brain structure or disease progression could overlap, we expect to further develop *J*_1_ in Equation (3) to leverage the useful information conveyed by the SNP correlations. Mathematically speaking, due to the interrelatedness among the SNPs, the learning vector (**b***_t_*)*_j_* should have certain correlations, where (**b***_t_*)*_j_* denotes the *j*th column of *B_t_*. Namely, the coefficient matrices *B_t_*(1 ≤ *t* ≤ *T*) should be of low rank. Given a general *n*-mode tensor 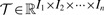 we denote 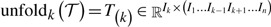 as the unfolding operation along its *k*th mode. Then we can achieve our goal by minimizing the rank of *B*_(1/_ = [*B*_1_, *B*_2_,...,*B_T_*] ∈ ℝ^*d*×(*c*×*T*/^ induced from 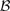, which leads to the following optimization problem:
(4)
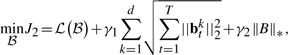

where || ||_*_ denotes the trace-norm of a matrix, and without ambiguity we drop the subscript of the matrix *B*_(1_*_/_* for notation brevity. Given a matrix *M* ∈ ℝ*^n×m^* and its singular values σ*_i_* (1 ≤ *i* ≤ min (*n,m*)), the trace-norm of *M* is defined as 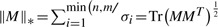. It has been shown that (Candes and Tao, 2010; Candès and Recht, 2009) the trace-norm is the best convex approximation of the rank-norm. Therefore, the third term of *J*_2_ in Equation (4) indeed minimizes the rank of the unfolded learning model 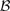, such that the correlations among the SNPs are captured. Due to its both capabilities for imaging marker selection and task correlation integration, we call *J*_2_ defined in Equation (4) as the proposed ‘task-correlated longitudinal sparse regression model’.

### 3.2 A new optimization algorithm and its global convergence

Because our new objective *J*_2_ is non-smooth, the problem in Equation (4) is difficult to solve in general. Some existing methods, such as LARS (Efron *et al.*, 2004), linear gradient search (Liu *et al.*, 2009), proximal (Beck and Teboulle., 2009) methods, can solve it, but not efficiently. Thus, in this subsection we derive a new efficient algorithm to solve *J*_2_ with rigorous proof of its global convergence.

Taking the derivative of *J*_2_ w.r.t *B_t_* and set it to zeros, we have:
(5)
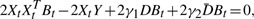

where *D* is a diagonal matrix with 
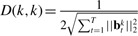
 and 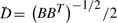. Thus, we can derive
(6)
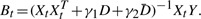

When the time *t* changes from 1 to *T*, we can compute *B_t_* (1≤ *t* ≤ *T* by Equation (6). Because *D* and 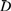 depend on *B* and can be seen as latent variables, we propose an iterative algorithm to obtain the global optimum solutions of 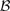 in Algorithm 1.


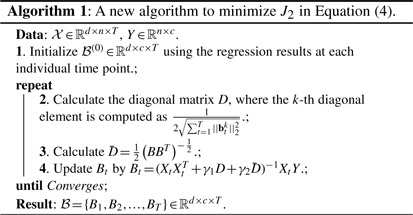


We summarize the convergence of Algorithm 1 as following.

Theorem 1. *Algorithm 1 monotonically decreases J_2_ in Equation (4) in each iteration, and converges to the globally optimal solution.*

***Proof***: In Algorithm 1, in each iteration we denote the updated *B_t_* as 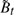 and the updated 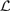 as 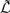. From step 4 we know that:
(7)
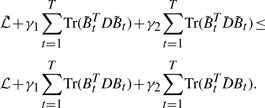


In each iteration, denote the updated *B* as 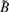 and the updated **b***_t_^k^* as 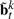, according to the definitions of *D* and 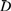, we can write
(8)
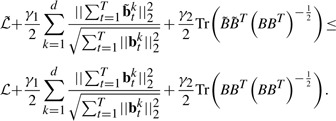


Following (Nie *et al.*, 2010, 2012), it can be verified that
(9)
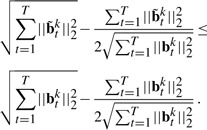

(10)
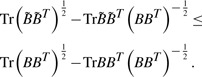


Adding the both sides of Equations (8–10) together, we obtain
(11)
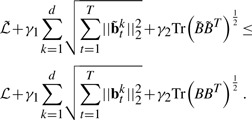


Thus, our algorithm decreases the objective value of Equation (4) in each iteration. When the objective value keeps unchange, Equation (5) is satisfied, i.e. the KKT condition of the objective is satisfied. Our algorithm reaches one of the optimal solution. Because our objective in Equation (4) is a convex problem, our Algorithm 1 will converge to one of the globally optimal solution.

**Computational analysis.** In the iteration loop of Algorithm 1, Step 2 is computationally trivial. Step 3 solves a singular value decomposition (SVD) problem, and Step 4 solves a system of linear equations, both of which, thereby the whole algorithm, are well studied in literature and can be solved very efficiently by existing numerical packages.

## 4 EXPERIMENTAL RESULTS AND DISCUSSIONS

In this section, we evaluate the proposed method by applying it to the ADNI cohort, where a wide range of imaging markers measured over a period of 2 years are examined and associated to SNPs that are relevant to AD. The goal is to discover a compact set of phenotypic imaging markers that are closely related to AD-sensitive genotypes encoded by SNPs.

### 4.1 Improved prediction of SNPs from longitudinal phenotypic imaging markers

We first evaluate the proposed method by applying it to the ADNI cohort to predict the SNPs of the participants from each of their two types of imaging phenotypes, i.e. VBM markers and FreeSurfer markers, tracked over four different time points, including BL and M06/M12/M24. Because some subjects of the ADNI cohort do not have complete imaging marker measurements over all the four time points, in our experiments we use the subject samples that have both SNPs data and complete imaging measurements. As a result, two subsets of ADNI subjects are included in our experiments, one for each type of imaging phenotypes, as detailed in Table 1.

**Table 1.** Numbers of participants in the experiments using two different types of imaging markers

Imaging phenotypes	# Total	# AD	# MCI	# HC
VBM	424	86	194	144
FreeSurfer	474	100	216	158

**Fig. 1.**
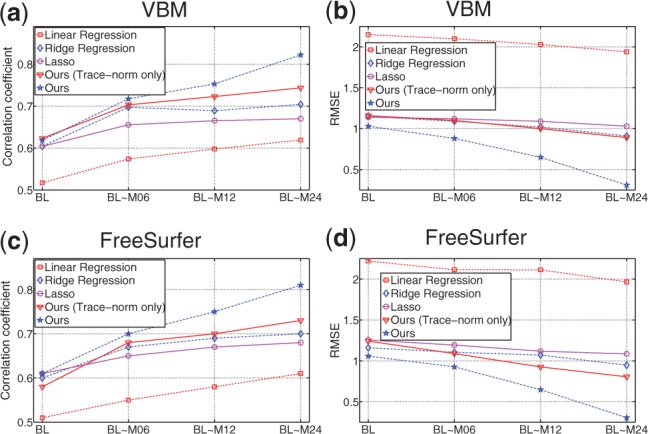
Regression performance with respect to the use of different number of longitudinal time points by three different methods

We compare the proposed method against its three close counterparts including multivariate linear regression (LR) method, ridge regression (RR) method and least absolute shrinkage and selection operator (Lasso) (Tibshirani, 1996) method. LR method is the most broadly used association model in both statistical learning and imaging genetics. RR method is the regularized version of LR model to avoid over-fitting. Lasso method replaces the squared *ℓ*_2_-norm regularization in RR method by the *ℓ*_1_-norm regularization, from which sparse results can be achieved (Tibshirani, 1996). Different to these compared methods, our new association model imposes structured sparsity via the tensor *ℓ*_2,1_-norm regularization for phenotypic marker selection and the trace-norm regularization for capturing the interrelationships among different SNPs. We implement two versions of the proposed method as follows. First, we implement our method by only imposing the trace-norm regularization, denoted as ‘Ours (Trace-norm only)’, which only makes use of the SNPs’ correlations, but does not select longitudinal imaging markers. Second, we implement the full version of the proposed method, denoted as ‘Ours’, which solves the problem in Equation (4). For measuring the regression performance of the five compared association models, we use a 5-fold cross-validation strategy by computing the Pearson's correlation coefficient (CORR) and the root mean square error (RMSE) between the predicted and the actual SNP values, which are reported in Figure 1.

As can be seen from Figure 1, if we only use the baseline data, the proposed method is reduced into a conventional multitask regression model, which appears as a matrix but not a tensor and achieves only the slightly better performance than the RR and Lasso methods. On the other hand, by using the longitudinal data, the performance of the proposed method is significantly improved, e.g. for predicting SNPs using the longitudinal data over all the four time points, the proposed (BL~ M24) method achieves the CORR of 0.793 and 0.812 and the RMSE of 0.314 and 0.301, respectively, which are much better than the case of using only the baseline data.

In addition, Figure 1 also shows that the usage of longitudinal data can improve the performances of all the LR, RR and Lasso methods, although the improvements are much less than the proposed method.

These results demonstrate the effectiveness of using longitudinal data for improved regression from imaging phenotypes to genotypes, especially by the proposed method, which has the capability to make use of the input data through longitudinal feature selection; and the integration of the interrelatedness among the SNPs.

### 4.2 Identification of longitudinal imaging markers

One primary goal of this study is to identify a subset of imaging phenotypes that are highly correlated to certain SNPs to capture important imaging genomic associations in AD research. Thus, we examine the phenotypic imaging markers identified by the proposed methods, which are relevant to the genotypes encoded by SNPs.

#### 4.2.1 Identified imaging markers with high AD risks

**Fig. 2.**
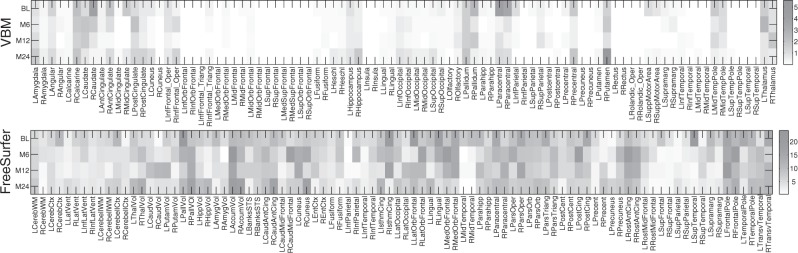
Weight maps of the association between imaging markers and the SNPs learned by the proposed method

**Fig. 3.**
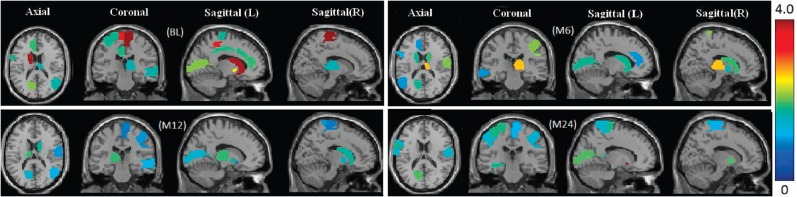
Visualization of top 10 VBM features selected by the proposed method at four different time points. The colors of the selected brain regions show the regression coefficients of the corresponding VBM markers

Shown in Figure 2 are the overall regression coefficients for all the VBM and FreeSurfer measures with respect to the 1224 SNPs used in this study. Because these SNPs are AlzGene candidates or proximal to the candidates, the results in Figure 2 can help identify SNP-relevant imaging phenotypes and have a potential to gain biological insights from gene to brain to symptoms. Besides, the top 10 selected VBM imaging features, as well as their association coefficients, are visualized in Figure 3 by mapping them onto the human brain.

A first glance at the association weigh maps shows that the selected imaging markers have clear patterns that span across all the four studied time points, which demonstrates that these phenotypic markers are longitudinally stable thus can serve as screening target over the course of AD progression. We also observe that hippocampal measures (LHippocampus, RHippocampus, LHippVol and RHippVol) are identified, which is in accordance with the fact that in the pathological pathway of AD, medial temporal lobe including hippocampus is firstly affected, followed by progressive neocortical damage (Braak and Braak, 1991; Delacourte *et al.*, 1999). The thickness measures of isthmus cingulate (LIsthmCing and RIsthmCing), frontal pole (LFrontalPole and RFrontalPole) and posterior cingulate gyrus (LPostCingulate and RPostCingulate) are also selected, which, again, is accordance with the fact that the GM atrophy of these regions is high in AD (Lehmann *et al.*, 2010; McEvoy *et al.*, 2009). In summary, the identified longitudinally stable markers strongly agree with the existing findings, which warrants the correctness of the discovered phenotype–genotype associations, and reveals the complex relationships among MRI measures, genetic variations and diagnosis status. This is of clear importance for theoretical research and clinical practices for a better understanding of AD mechanism.

#### 4.2.2 Case studies: markers identified for rs423958-APOE and rs11136000-CLU

**Fig. 4.**
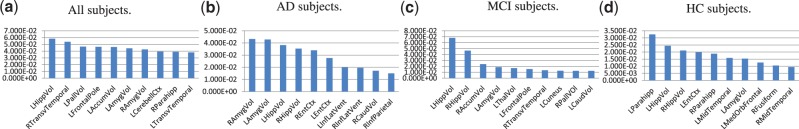
Top 10 FreeSurfer markers identified for rs423958-APOE.

**Fig. 5.**
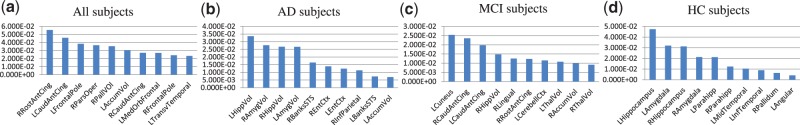
Top 10 FreeSurfer markers identified for rs11136000-CLU

We provide two case studies to show the top 10 FreeSurfer markers associated with two major AD risk SNPs: rs423958-APOE and rs11136000-CLU. We explore the associations between the FreeSurfer markers and the two SNPs in four different subject groups induced from the ADNI data, i.e. the groups of All, AD, MCI and HC participants, respectively. The number of the subjects in each group is available in Table 1. We select the imaging markers by their average regression coefficients over all the four time points. The top 10 FreeSurfer markers relevant to rs423958-APOE and their regression coefficients are shown in Figure 4 and those relevant to rs11136000-CLU are shown in Figure 5. From Figure 4 we can see that most of the top 10 FreeSurfer markers for rs423958-APOE in the four different testing groups are well-known AD-sensitive phenotypes, such as hippocampal volume in All, AD, MCI and HC groups; amygdala volume in All, AD, MCI and HC groups; accumbens volume in All and MCI groups and entorhinal cortex thickness in AD and HC groups; Similar patterns are also observed for rs11136000-CLU, as shown in Figure 5. Although data are not shown due to space limit, our VBM analyses have also yielded similar results. The complete imaging marker identification results by our method for both VBM and FreeSurfer markers on the top 10 identified SNPs are available at the author's website at http://ranger.uta.edu/%7eheng/imgsnp/. These results have again demonstrated the promise of the proposed method in terms of its capability to identify imaging markers relevant to AD-sensitive SNPs.

## 5 CONCLUSIONS

Elucidating the associations between longitudinal phenotypic imaging markers and AD sensitive SNPs is of important value for both scientific research and clinical practice. In this article, we presented a new task-correlated longitudinal sparse regression method to identify longitudinal imaging markers to AD-relevant SNPs. In our newly proposed regression model, we imposed a tensor *ℓ*_2,1_-norm regularization extended from the standard matrix *ℓ*_2,1_-norm to capture the temporal patterns in the longitudinal data over all the tasks of interest, and meanwhile imposed the trace-norm regularization onto the unfolded coefficient tensor such that the interrelatedness among the SNPs during the progression of AD conversion is addressed. Due to the additional time dimension of the input data and the non-smoothness of the tensor *ℓ*_2,1_-norm and trace-norm, solving the formulated objective of our new method was very challenging. Therefore, we presented an efficient iterative algorithm and rigorously proved its convergence to the global optimum. We applied the proposed method to the ADNI cohort and evaluated it in both SNPs prediction and longitudinal imaging marker identification. The clearly improved regression performance in the prediction and highly suggestive imaging markers selected by our new method have validated its effectiveness.

*Funding:* This research was supported by the National Science Foundation Grant (NSF) CCF-0830780, CCF-0917274, DMS-0915228 and IIS-1117965 at UTA; and by NSF IIS-1117335, NIH UL1 RR025761, U01 AG024904, NIA RC2 AG036535, NIA R01 AG19771 and NIA P30 AG10133-18S1 at IU.

Data collection and sharing for this project was funded by the ADNI (National Institutes of Health Grant U01 AG024904). ADNI is funded by the National Institute on Aging, the National Institute of Biomedical Imaging and Bioengineering, and through generous contributions from the following: Abbott; Alzheimer's Association; Alzheimer's Drug Discovery Foundation; Amorfix Life Sciences Ltd.; AstraZeneca; Bayer HealthCare; BioClinica, Inc.; Biogen Idec Inc.; Bristol-Myers Squibb Company; Eisai Inc.; Elan Pharmaceuticals Inc.; Eli Lilly and Company; F. Hoffmann-La Roche Ltd and its affiliated company Genentech, Inc.; GE Healthcare; Innogenetics, N.V.; Janssen Alzheimer Immunotherapy Research & Development, LLC.; Johnson & Johnson Pharmaceutical Research & Development LLC.; Medpace, Inc.; Merck & Co., Inc.; Meso Scale Diagnostics, LLC.; Novartis Pharmaceuticals Corporation; Pfizer Inc.; Servier; Synarc Inc. and Takeda Pharmaceutical Company. The Canadian Institutes of Health Research is providing funds to support ADNI clinical sites in Canada. Private sector contributions are facilitated by the Foundation for the National Institutes of Health (www.fnih.org). The grantee organization is the Northern California Institute for Research and Education and the study is coordinated by the Alzheimer's Disease Cooperative Study at the University of California, San Diego. ADNI data are disseminated by the Laboratory for Neuro Imaging at the University of California, Los Angeles. This research was also supported by NIH grants P30 AG010129, K01 AG030514 and the Dana Foundation.
